# Treatment of children with acute upper respiratory tract infection (wind-heat pattern) with Yin Hu Gan Mao San: a prospective real-world cohort study

**DOI:** 10.3389/fphar.2026.1838314

**Published:** 2026-07-02

**Authors:** Jialin Li, Teng Huang, Lihua Ning, Gen Lu, Chunying Tan, Songhua Fang, Shan Hua, Zhenkun Zhang, Zhou Fu, Ping He, Wei Hou, Ya Zhou, Tao Yang, Hongfang Luo, Huiyuan Zhang, Jun Tang, Yun Liu, Rongchang Shao, Yourui Du, Fujian Liao, Xueming Li, Yanhong Jia, Ming Yan, Hui Zhen, Junping Ding, Hongping Chen, Youjia Xu, Baoping Xu

**Affiliations:** 1 China National Clinical Research Center of Respiratory Diseases Respiratory Department of Beijing Children’s Hospital Capital Medical University National Center for Children’s Health, Beijing, China; 2 Pediatrics Department, Guangdong Provincial Hospital of Chinese Medicine, Guangzhou, Guangdong, China; 3 Pediatrics Department, The Fourth Hospital of Baotou, Baotou, Inner Mongolia, China; 4 Respiratory Department, Guangzhou Women and Children’s Medical Center, Guangzhou, Guangdong, China; 5 Pediatrics Department, Liaoning Maternal and Child Health Hospital, Shenyang, Liaoning, China; 6 Emergency Department, Shenzhen Children’s Hospital, Shenzhen, Guangdong, China; 7 Respiratory Medicine, Anhui Provincial Children’s Hospital, Hefei, Anhui, China; 8 Respiratory Medicine, Xuzhou Children’s Hospital, Xuzhou, Jiangsu, China; 9 Respiratory Department, Children’s Hospital of Chongqing Medical University, Chongqing, China; 10 Respiratory Department, Yunnan Provincial Hospital of Traditional Chinese Medicine, Kunming, Yunnan, China; 11 Respiratory Department, The Second Affiliated Hospital of Xi’an Jiaotong University, Xian, Shaanxi, China; 12 Respiratory Department Medical University Affiliated Shengjing Hospital Dalian Hospital, Dalian, Liaoning, China; 13 Respiratory Department, Kunming Yanan Hospital, Kunming, Yunnan, China; 14 Respiratory Department, The Second Affiliated Hospital of Guizhou Medical University, Kaili City, Guizhou, China; 15 Respiratory Department, The Hospital of Shanxi University of Chinese Medicine, Taiyuan Shanxi, China; 16 Respiratory Department, Guangzhou Liwan District Maternal and Child Health Hospital, Guangzhou, Guangdong, China; 17 Respiratory Department, Urumqi First People’s Hospital, Urumqi, Xinjiang, China; 18 Respiratory Department, Ezhou Central Hospital, Ezhou, Hubei, China; 19 Respiratory Department, Yuncheng Traditional Chinese Medicine Hospital, Yuncheng Shanxi, China; 20 Respiratory Department, Chongqing Yongchuan District Hospital of Traditional Chinese Medicine, Chongqing, China; 21 Respiratory Department, Handan Hospital of Traditional Chinese Medicine, Handan, Hebei, China; 22 Pediatric Medicine, Shan Xi Fen Yang Hospital, Fenyang Shanxi, China; 23 Respiratory Department, Jinzhou District First People’s Hospital, Dalian Liaoning, China; 24 Respiratory Disease Research Committee of China Association of Traditional Chinese Medicine, Beijing, China; 25 Harbin Kangsaisi Medical Technology Development Co., Ltd., Harbin Heilongjiang, China; 26 Beijing Yaohai Ningkang Pharmaceutical Technology Co., Ltd., Beijing, China

**Keywords:** acute upper respiratory tract infection, children, cohort study, real-world study, wind-heat pattern, Yin Hu Gan Mao San

## Abstract

**Background:**

Yin Hu Gan Mao San (YHGMS) is a traditional Chinese herbal preparation derived from Yin Qiao San, used for topical application to treat wind-heat colds. This study aims to evaluate the clinical efficacy and safety of YHGMS in children with acute upper respiratory tract infection (AURTI) of wind-heat pattern.

**Methods:**

This prospective, real-world cohort study enrolled 999 children with AURTI from 23 hospitals from May 2022 to June 2023. Participants were naturally divided into an exposed group (n = 671) that received YHGMS and a non-exposed group (n = 328) that did not receive YHGMS or any other traditional Chinese medicine. Propensity score matching was used to balance baseline characteristics. Primary outcomes included clinical recovery rate and time to recovery. Secondary outcomes included time to normal temperature, symptom resolution rates, and safety profile.

**Results:**

After propensity score matching, the YHGMS group demonstrated significantly higher recovery rates on day 3 (52.64% vs*.* 36.09%), day 5 (81.00% vs*.* 68.81%), and day 7 (94.72% vs*.* 87.64%) (all *P* < 0.0001). Median recovery time (3.0 vs*.* 4.0 days) and time to normal temperature (1.0 vs*.* 2.0 days) were significantly shorter in the YHGMS group (both *P* < 0.0001). Resolution rates of individual symptoms were consistently higher in the YHGMS group. The incidence of adverse reactions was low (1.51%), comprising only mild, self-limiting local skin reactions.

**Conclusion:**

YHGMS was associated with faster clinical recovery and symptom resolution in children with AURTI (wind-heat pattern), without significant safety concerns. As monotherapy, YHGMS showed better outcomes than chemical medicine alone; in combination, it also accelerated resolution of clinical symptoms and recovery.

## 1 Introduction

Acute upper respiratory tract infection (AURTI) is a common condition in children, encompassing acute inflammation of the nasal cavity, pharynx and larynx, including the common cold, viral pharyngitis, laryngitis, herpangina, pharyngoconjunctival fever, and bacterial pharyngotonsillitis (Chinese Medical Association et al., 2019). AURTI is typically self-limited and has a good prognosis, but its high incidence and potential for complications (e.g., otitis media, bronchitis, pneumonia) impose a considerable burden on healthcare systems and families worldwide (Chinese Medical Association et al., 2019; Hawke et al., 2022).

Viruses account for 70%–80% of AURTI cases, including influenza, parainfluenza, respiratory syncytial virus, adenovirus, rhinovirus, echovirus, coxsackievirus, measles virus and rubella virus. Bacteria account for the remaining 20%–30%, with *Hemolytic streptococcus* being the most common, followed by *Haemophilus influenzae*, *Streptococcus pneumoniae* and *Staphylococcus aureus*, and occasionally Gram-negative bacteria (Jain N et al., 2001). Despite the predominantly viral etiology, antibiotics are frequently overprescribed for AURTI in outpatient settings, especially in China, contributing to the global threat of antimicrobial resistance (AMR) (Ma and Shen, 2021). Current international guidelines discourage routine antibiotic use and recommend symptomatic treatments, including rest, hydration, antipyretics, and nasal decongestants (Uyeki et al., 2019; Chinese Medical Association et al., 2020). However, effective symptom relief remains an unmet need. Common over-the-counter (OTC) medications such as decongestants, antihistamines, and analgesics have shown limited efficacy in young children, and their use may be associated with adverse effects including drowsiness, dry mouth, and gastrointestinal upset (De Sutter et al., 2022).

Traditional Chinese medicine (TCM) has been used for centuries to treat AURTI. Several TCM formulas have shown benefits in reducing fever duration and alleviating cough, nasal congestion, rhinorrhea and sore throat in children with AURTI (Qiu et al., 2014; Ouyang et al., 2022). Nevertheless, oral administration of TCM decoctions is often limited by poor palatability and low adherence in pediatric patients. Acupoint application therapy, which delivers herbal extracts transdermally, avoids the first-pass metabolism, reduces gastrointestinal side effects, and improves compliance (Liu and Lv, 2004; Xiong et al., 2021). Therefore, a well-standardized, externally applied TCM preparation could be a valuable addition for pediatric AURTI, particularly in the context of antimicrobial stewardship.

Yin Hu Gan Mao San (YHGMS, SFDA approval number: Z20026714) is a modern acupoint patch derived from the classical formula Yin Qiao San. YHGMS comprises 12 botanical drugs, including *Baeckea frutescens* L.L. [*Myrtaceae*], *Eucalyptus robusta* Sm. [*Myrtaceae*], *Lonicera japonica* Thunb. [*Caprifoliaceae*], *Forsythia suspensa* (Thunb.) Vahl [*Oleaceae*], *Artemisia annua* L. [*Asteraceae*], *Schizonepeta tenuifolia* Briq. [*Lamiaceae*], *Mentha haplocalyx* Briq. [*Lamiaceae*], *Bupleurum chinense* DC. [*Apiaceae*], *Pogostemon cablin* (Blanco) Benth. [*Lamiaceae*], *Artemisia argyi* H. Lév. & Vaniot [*Asteraceae*], *Platycodon grandiflorum* (Jacq.) A. DC. [*Campanulaceae*], and *Citrus reticulata* Blanco [*Rutaceae*]. Pharmacological studies have demonstrated that YHGMS exhibits antibacterial, anti-inflammatory, antiviral, antipyretic, and antitussive effects *in vitro* and in animal models (Chen et al., 2003). In addition, volatile oils from several individual botanical drugs (e.g., *Baeckea frutescens*, *E. robusta* leaf, *A. annua*, and *F. suspensa)* have been shown to possess anti-inflammation and antibacterial activities *in vitro*; volatile oils from *M. haplocalyx*, *S. tenuifolia*, *P. cablin*, *A. argyi*, and *C. reticulata* have been reported to have transdermal absorption-enhancing properties as well as antibacterial, anti-inflammatory, temperature reduction, analgesia (Liu and Mao, 2007; Luo et al., 2007; Bai et al., 2008; Shen and Li, 2008; Li et al., 2011; Yu et al., 2013; Song, 2014; Tian et al., 2018; Zheng et al., 2018; Wang et al., 2019; Jia et al., 2020; Zheng et al., 2020; Shangguan and Huang, 2021). Furthermore, components isolated from these botanical drugs have significant inhibitory effects against respiratory syncytial virus and parainfluenza virus *in vitro* (Tian et al., 2004; Ma et al., 2006).

Despite the theoretical advantages of acupoint application, the clinical effectiveness and safety of YHGMS in children with AURTI have not been systematically evaluated in a real-world setting. To address this gap, we conducted a prospective, multicenter, real-world cohort study to assess whether adding YHGMS to conventional care is associated with faster clinical recovery, earlier fever resolution, and better symptom relief compared with conventional care alone. Additionally, the study aimed to explore age-appropriate dosing and to document the safety profile of YHGMS in a large pediatric population. We hypothesized that YHGMS would shorten illness duration and reduce symptom burden without increasing the risk of adverse events.

## 2 Materials and methods

### 2.1 Study design and population

This is a prospective, observational, multi-center cohort study. A total of 999 children under 18 years of age with AURTI of the wind-heat pattern according to TCM were enrolled between May 2022 and June 2023 at 23 hospitals using a clinical quota sampling method. Participants were naturally allocated into an exposed group (who received YHGMS) and a non-exposed group (who did not receive YHGMS or any other TCM) based on routine clinical practice, with a target allocation ratio of approximately 2:1. The decision to prescribe YHGMS was made by the treating physician, taking into account the child’s condition and parental preference. No interventions were administered on the children before enrollment. After enrollment, both groups received symptomatic treatment as needed (e.g., antipyretics, expectorants, nasal decongestants) according to *Expert Consensus on the Diagnosis and Treatment of Common Cold in Chinese Children (2013)* (Lu et al., 2013), while the exposed group additionally received YHGMS. Neither group received any other TCM during the observation period.

The diagnostic criteria for AURTI and wind-heat pattern in TCM were based on *Guidelines for the Diagnosis and Treatment of Common Pediatric Diseases of Traditional Chinese Medicine (2012)* and *Technical Guidelines for the Design and Evaluation of Clinical Trials of New Chinese Medicines for Common Pediatric Diseases Acute Upper Respiratory Tract Infections in Children* (China Association of Chinese Medicine, 2012; Society of Pediatrics, 2013), as detailed in Supplementary Table S1. To ensure diagnostic consistency across the 23 participating centers, all investigators underwent standardized training in the wind-heat pattern criteria. Cases with a confirmed diagnosis of coronavirus disease 2019 (COVID-19) or influenza (based on nucleic acid testing) were excluded.

This study was conducted in accordance with the *Declaration of Helsinki* and the *International Ethical Guidelines for Health-related Research Involving Humans.* The protocol was approved by the Ethics Committee of Guangdong Provincial Hospital of Traditional Chinese Medicine (BF2022-077-01). Written informed consent was obtained from the legal guardians of all enrolled children; assent was also obtained from children over 8 years of age where applicable. This study was registered in the Chinese Clinical Trial Registry (ChiCTR2300075215).

### 2.2 Chemical profiling and quality control

YHGMS is manufactured by Guangxi Yuanantang Pharmaceutical Co., Ltd. under Good Manufacturing Practice (GMP) conditions. All raw materials were sourced from GAP-certified cultivation bases. The botanical drugs were authenticated by the manufacturer, and voucher specimens are deposited at its quality control department. The drug-to-extract ratio is approximately 14,908 g of crude herbs to 1,000 g of powder and 90.2 mL of medicinal oil. Three production batches were used in this study, and their quality consistency was verified by a gas chromatography fingerprint method (Li et al., 2025).

The quality of YHGMS complies with the national drug standard WS-11140 (ZD-1140)-2002-2012Z, which includes thin layer chromatography identification and high performance liquid chromatography quantification of chlorogenic acid. The ConPhyMP checklists are included in Supplementary Materials S1a, S2.

### 2.3 Medication regimen of YHGMS

Acupoint selection: The primary application points were Dazhui (DU-14, a classic acupoint for expelling exterior pathogens and reducing fever) or Shenque (RN-8, believed to strengthen vital Qi and regulate immune function), either alone or in combination with additional points as the discretion of the prescribing physician.

Application method: The medicinal oil was poured into the powder packet, and then mixed, and the patch was applied to the selected acupoint.

Application dose: One patch application per acupoint per day.

Application duration: The patch was left in place for varying durations depending on the child’s age: 4 h for children aged 0–2 years, 6 h for 3–4 years, 8 h for 5–6 years, 10–12 h for 7–12 years, and 12–24 h for children over 12 years. These durations were based on the manufacturer’s instructions and standard clinical practice.

### 2.4 Outcomes

The maximum observation period was 7 days from cohort entry. If the child achieved clinical recovery within 7 days, the study treatment could be discontinued at the physician’s discretion based on the child’s condition. All data were recorded at baseline (cohort entry) and throughout the observation period using standardized case report forms and diary cards. The dataset included demographic characteristics, treatment details, laboratory tests, chest radiological findings, efficacy outcomes and safety outcomes.

The primary efficacy outcomes were clinical recovery rate and time to clinical recovery. Clinical recovery was defined as normal body temperature (<37.3 °C, axillary), resolution of all major symptoms (nasal congestion, rhinorrhea, sneezing, cough, and sore throat), no interference with daily activities or sleep, and sustained for at least 24 h. Secondary efficacy outcomes included time to normal temperature, use of antipyretic drugs (acetaminophen or ibuprofen), resolution rates of individual symptoms at days 3, 5, and 7. Safety outcomes included the incidence of adverse events (AEs) and local skin reactions.

### 2.5 Statistical analysis

The statistical analyses were conducted using SAS version 9.4. Based on a 7-day clinical recovery rate of 85% in the non-exposed group, we assumed that the exposed group would achieve a 10% absolute improvement. Under a two-sided significance level of 0.05% and 80% power, with an allocation ratio of 2:1 (exposed: non-exposed), a minimum of 238 exposed and 119 non-exposed participants was required to detect such a difference. Considering the potential variations in baseline demographics, disease severity, treatment patterns, and sampling error inherent in real-world studies, we ultimately enrolled 666 exposed and 333 non-exposed participants, resulting in a total of 999 children.

Quantitative data were presented as mean ± SD (normal distribution) or median duration and quartile (non-normal distribution), and compared using Student’s t-test (normal distribution) or Wilcoxon rank-sum test (non-normal distribution). Qualitative data were presented as counts (N) and percentages (%) and compared using the chi-square test or Fisher’s exact test. Time-to-event data (clinical recovery time and time to normal temperature) were summarized using the Kaplan–Meier method and compared using the log-rank test, with results presented as median (Q1, Q3).

Subgroup analyses were performed to evaluate the influence of age, disease duration, presence of fever, and different treatment combinations (e.g., YHGMS alone vs. YHGMS plus chemical drugs) on clinical outcomes. In order to minimize confounding by indication and other baseline imbalances, propensity score matching (PSM) was employed as the primary adjustment method. Covariates were selected *a priori* based on their clinical relevance and potential to influence both treatment assignment and outcomes, including age, sex, presence of fever, duration of disease, history of recurrent respiratory tract infection (RRTI), disease severity, use of AURTI medication in the past week, use of compound preparations, use of antibiotics or antivirals, and use of antipyretics. Propensity scores were calculated for each participant. One-to-one nearest-neighbor matching was performed using a caliper of 0.2 times the standard deviation of the logit of the propensity score. Balance was assessed by comparing baseline characteristics between matched groups. Other adjustment methods were also employed for sensitivity analysis. No imputation was performed due to minimal missing data.

## 3 Results

A total of 999 children with AURTI were screened, and 990 (99.1%) were enrolled in 23 participating sites, including 663 in the exposed group and 327 in the non-exposed group.

### 3.1 Baseline characteristics

Gender distribution was similar between the two groups (54.00% vs. 54.13% male). The mean age was 3.73 ± 2.99 years in the exposed group and 4.14 ± 3.04 years in the non-exposed group (*P* = 0.0280), but there was no significant difference in age group distribution (*P* = 0.0716). Proportion of severe AURTI was significantly lower in the exposed group than in the non-exposed group (29.26% vs. 38.84%, *P* = 0.0025). About two-thirds of the children experienced fever, with a higher incidence of fever observed in the exposed group (69.68% vs. 63.00%, *P* = 0.0347). Before cohort entry, about 20% of children in both groups had used drugs to treat AURTI. In addition, no significant differences were observed between the two groups in BMI, history of RRTIs, duration of disease or duration of fever. After 1:1 PSM, the baseline characteristics of the two groups exhibited satisfactory comparability (Table 1).

**TABLE 1:** Baseline characteristics in original sample and in propensity score-matched sample.

Indicators	Original sample			Matched sample		
	Exposed group (n = 663)	Non-exposed group (n = 327)	*P* value	Exposed group (n = 288)	Non-exposed group (n = 288)	*P* value
Gender [n (%)]						
Male	358 (54.00)	177 (54.13)	0.9689	161 (55.90)	155 (53.82)	0.6752
Female	305 (46.00)	150 (45.87)	​	127 (44.10)	133 (46.18)	​
Age (year)	3.73 ± 2.99	4.14 ± 3.04	**0.0280**	4.14 ± 3.13	4.21 ± 3.04	0.7840
Age group [n (%)]						
0 years∼	200 (30.17)	82 (25.08)	0.0716	68 (23.61)	69 (23.96)	0.9059
2 years∼	182 (27.45)	78 (23.85)	​	70 (24.31)	67 (23.26)	​
4 years∼	135 (20.36)	76 (23.24)	​	75 (26.04)	70 (24.31)	​
6 years∼	146 (22.02)	91 (27.83)	​	75 (26.04)	82 (28.47)	​
Height (cm)	100.78 ± 23.06	104.41 ± 24.46	**0.0300**	103.18 ± 23.33	103.69 ± 23.84	0.7952
Weight (kg)	17.16 ± 9.25	18.57 ± 10.50	**0.0350**	18.42 ± 10.22	18.58 ± 9.92	0.8531
BMI (kg/m^2^)	16.44 ± 2.73	16.54 ± 2.81	0.8696	16.39 ± 2.84	16.45 ± 2.71	0.8074
History of RRTIs [n (%)]	56 (8.45)	19 (5.81)	0.1404	23 (8.00)	18 (6.25)	0.5168
Duration (h)	36.73 ± 23.30	36.83 ± 19.04	0.3590	35.15 ± 19.83	34.67 ± 17.99	0.7631
Duration group [n (%)]						
0 h∼	129 (19.46)	46 (14.07)	0.0820	57 (19.79)	46 (15.97)	0.3180
24 h∼	262 (39.52)	146 (44.65)	​	120 (41.67)	136 (47.22)	​
48 h∼	272 (41.03)	135 (41.28)	​	111 (38.54)	106 (36.81)	​
Fever [n (%)]	462 (69.68)	206 (63.00)	**0.0347**	191 (66.32)	198 (68.75)	0.5927
Fever duration (h)	26.29 ± 16.73	26.65 ± 14.81	0.5256	25.72 ± 15.78	26.38 ± 14.83	0.6712
Fever duration [n (%)]						
0 h∼	159 (34.42)	59 (28.64)	0.1632	66 (22.92)	59 (20.49)	0.5109
24 h∼	193 (41.77)	102 (49.51)	​	84 (29.17)	98 (34.03)	​
48 h∼	110 (23.81)	45 (21.84)	​	42 (14.58)	42 (14.58)	​
Use of AURI drug within 1 week [n (%)]	147 (22.17)	78 (23.85)	0.5527	60 (20.83)	72 (25.00)	0.2751

Bold values indicate *P* < 0.05.

### 3.2 Clinical recovery

**FIGURE 1:**
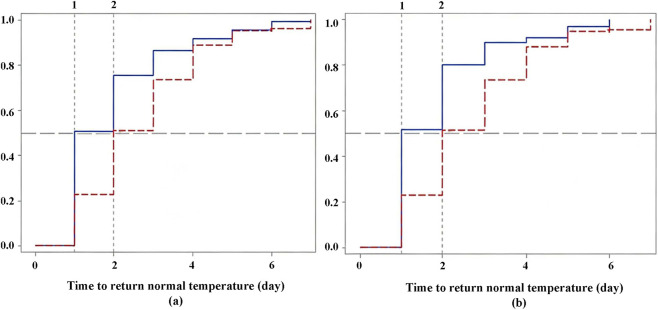
Kaplan-Meier curve of clinical recovery time of exposed group and non-exposed group **(a)** Original sample; **(b)** Matched sample after propensity score matching. Blue solid line: exposed group; red dashed line: non-exposed group.

The clinical recovery rate and Kaplan-Meier curves of children in the two groups are shown in Table 2 and Figure 1. In the original cohort, the clinical recovery rates in the exposed group at day 3, 5 and 7 were significantly higher than those in the non-exposed group with risk differences of 16.55% (95% CI: 10.11%–23.00%), 12.19% (95% CI: 6.35%–18.03%), and 7.26% (95% CI: 3.29%–11.23%), respectively (all *P* < 0.0001). The median recovery time was 3 days (IQR: 3–5) in the exposed group and 4 days (IQR: 3–6) in the non-exposed group. Log-rank test showed a significant difference between the two groups (*P* < 0.05), and Cox regression yielded a hazard ratio of 1.333 (95% CI: 1.163–1.529) for the exposed group relative to the non-exposed group.

**TABLE 2:** Analysis of clinical recovery of upper respiratory tract infection [n (%)].

Indicators	Original cohort				Matched sample			
	Exposed (n = 663)	Non-exposed (n = 327)	RD (95% CI)	*P* value	Exposed (n = 288)	Non-exposed (n = 288)	RD (95% CI)	*P* value
Rate of recovery in 3 days	349 (52.64)	118 (36.09)	16.55 (10.11–23.00)	**<0.001**	151 (52.43%)	102 (35.42%)	17.01 (9.03–25.00)	**<0.001**
Rate of recovery in 5 days	537 (81.00)	225 (68.81)	12.19 (6.35–18.03)	**<0.001**	231 (80.21%)	200 (69.44%)	10.76 (3.73–17.80)	**<0.001**
Rate of recovery in 7 days	628 (94.72)	286 (87.46)	7.26 (3.29–11.23)	**<0.001**	276 (95.83%)	249 (86.46%)	9.38 (4.80–13.95)	**<0.001**
Clinical recovery time (days)	3.0 (3.0–5.0)	4.0 (3.0–6.0)	1.33 (1.16–1.53)^*^	**<0.001**	3.0 (3.0,5.0)	4.0 (3.0,6.0)	1.38 (1.16–1.63)*	**<0.001**

* The effect size of Clinical Recovery Time is Hazard Ratio (95% CI). Bold values indicate *P* < 0.05.

After PSM, the results were consistent. The recovery rates at days 3, 5, and 7 were 52.43% vs. 35.42% (risk difference 17.01%, 95% CI: 9.03%–25.00%), 80.21% vs. 69.44% (risk difference 10.76%, 95% CI: 3.73%–17.80%), and 95.83% vs. 86.46% (risk difference 9.38%, 95% CI: 4.80%–13.95%), respectively (all *P* < 0.001). The median recovery time remained 3 days (IQR: 3–5) in the exposed group and 4 days (IQR: 3–6) in the non-exposed group, with a hazard ratio of 1.38 (95% CI: 1.16–1.63) and a log-rank *P* < 0.001.

### 3.3 Proportion and time of resolution of each symptom and sign

**FIGURE 2:**
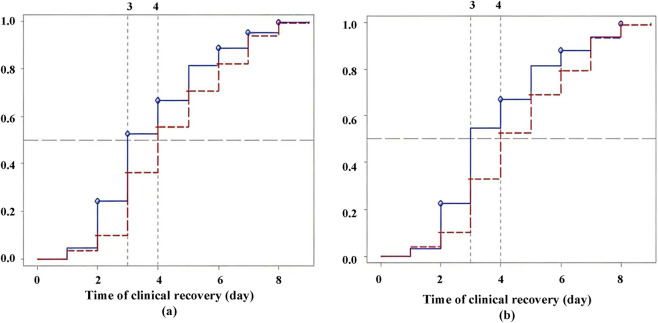
Kaplan-Meier curve of time to return normal temperature of exposed group and non-exposed group. **(a)** Original sample; **(b)** Matched sample after propensity score matching. Blue solid line: exposed group; red dashed line: non-exposed group.

For children with fever at baseline, the median time to normal temperature was 1 day (IQR: 1–2) in the exposed group and 2 days (IQR: 2–4) in the non-exposed group. Log-rank test indicated a significant difference between the two groups (*χ* ^*2*^ = 27.7828, *P* < 0.0001), with a hazard ratio of 1.536 (95% CI: 1.301–1.814) from the Cox regression model. After PSM, the hazard ratio was 1.443 (95% CI: 1.179–1.767) (Figure 2). The utilization rates of antipyretic drugs were 18.70% in the exposed group and 22.02% in the non-exposed group, with a risk difference of −3.32% (95% CI: −8.70%–2.07%, *P* = 0.2182). In the matched cohort, the rates were 18.06% (52/288) and 24.65% (71/288), respectively (risk difference −6.60%, 95% CI: −13.27%–0.07%, *P* = 0.053).

Tables 3, 4 illustrates the resolution rates of individual TCM symptoms. For fever resolution by day 3, the exposed group had a significantly higher rate than the non-exposed group (86.03% vs. 73.79%; risk difference 12.24%, 95% CI: 5.61%–18.87%, *P* = 0.0001). Similarly, for cough and nasal congestion at day 3, the exposed group showed higher resolution rates (53.98% vs. 43.28%; risk difference 10.70%, 95% CI: 2.92%–18.48%, *P* = 0.0075; and 65.50% vs. 52.36%; risk difference 13.14%, 95% CI: 5.75%–20.53%, *P* = 0.0004, respectively). By day 5, the fever resolution rate exceeded 95% in both groups, with no significant difference. For other symptoms, the resolution rates in the exposed group were significantly higher than those in the non-exposed group at any time point (all *P* < 0.05), and results were consistent. Detailed data can be found in Supplementary Table S2.

**TABLE 3:** Resolution rate of each symptom at different time nodes (%) - Original sample.

Symptom	Day 3		Day 5		Day 7	
	Exposed	Non-exposed	Exposed	Non-exposed	Exposed	Non-exposed
Fever (458 vs. 206)^*^	86.03	73.79	95.20	95.15	99.78	99.03
Stuffy (513 vs. 254)	65.50	52.36	85.58	72.05	91.03	84.65
Runny nose (538 vs. 265)	63.57	48.68	80.11	66.04	89.22	79.25
Sneeze (443 vs. 216)	72.91	58.80	86.23	73.61	94.81	81.48
Sore throat (559 vs. 283)	68.16	60.42	83.54	76.33	88.01	81.98
Cough (452 vs. 238)	53.98	43.28	73.67	60.92	81.86	70.17
Wind aversion (275 vs. 137)	67.64	54.74	91.27	73.72	98.18	91.97
Sweat (517 vs. 264)	59.77	42.80	82.40	69.32	91.10	80.30
Expectoration (322 vs. 172)	59.94	46.51	72.67	57.56	86.02	72.09
Flush face (416 vs. 199)	70.67	56.28	88.70	76.38	95.91	84.42
Dysphoria (339 vs. 136)	58.11	40.44	83.19	66.18	94.99	79.41
Headache (137 vs. 95)	71.53	54.74	90.51	81.05	97.08	90.53
Thirst (404 vs. 205)	51.98	34.63	79.70	60.49	90.84	74.63
Dark urine (556 vs. 263)	39.39	32.32	66.55	51.71	78.78	68.06

*Numbers in parentheses indicate the number of children with that symptom in the exposed and non-exposed groups, respectively.

**TABLE 4:** Resolution rate of each symptom at different time nodes (%) - matched sample.

Symptom	Day 3		Day 5		Day 7	
	Exposed	Non-exposed	Exposed	Non-exposed	Exposed	Non-exposed
Fever (191 vs. 198)^*^	85.42	73.61	94.10	95.14	100.00	98.96
Stuffy nose (231 vs. 225)	66.67	52.43	84.03	72.92	89.58	86.11
Purulent rhinorrhea (241 vs. 230)	62.50	49.65	82.29	65.97	89.24	80.56
Sneeze (189 vs. 187)	71.88	60.07	85.76	74.31	94.10	82.29
Sore throat (236 vs. 244)	68.06	62.50	82.64	78.82	87.50	81.60
Cough (193 vs. 203)	54.86	43.75	75.00	65.97	80.56	72.92
Wind aversion (123 vs. 134)	65.28	54.51	90.97	74.31	98.26	93.40
Sweat (215 vs. 227)	60.76	47.22	81.25	70.83	92.71	82.99
Expectoration (141 vs. 141)	67.01	50.35	80.21	60.76	89.93	77.78
Flush face (185 vs. 174)	72.22	60.76	89.93	80.21	96.53	89.58
Dysphoria (132 vs. 118)	56.94	46.18	81.94	70.14	94.10	85.42
Headache (68 vs. 93)	66.67	53.82	87.85	77.78	98.61	89.58
Thirst (186 vs. 184)	48.96	35.07	79.51	58.33	89.93	73.96
Dark urine (246 vs. 227)	43.06	34.38	65.28	51.39	77.08	69.10

*Numbers in parentheses indicate the number of children with that symptom in the exposed and non-exposed groups, respectively.

### 3.4 Subgroup analysis

Given the exploratory nature of subgroup analyses and the increased risk of type I error due to multiple comparisons, these findings should be interpreted cautiously.

#### 3.4.1 Effect of YHGMS and other drugs

Based on whether the patients in the exposed group were treated with chemical medicine, they were divided into two groups. Only using YHGMS group, refers to not taking any chemical drugs, and YHGMS combined group, refers to the use of YHGMS while receiving chemical medicine treatment. Effects of the two groups and chemical medicine-only group (chemical group) were compared. YHGMS combined group demonstrated the highest clinical recovery rate of AURTI on day 3 (57.27%), followed by only using YHGMS group (47.65%), and the lowest in chemical group (36.09%). The recovery rates on day 5 were 84.88%, 76.80%, and 68.81% respectively, remaining consistent with those on day 3. Although the differences between these groups narrowed, they remained statistically significant. By the end of day 7, the recovery rates of groups treated with YHGMS had reached over 90%, a statistically significant difference compared to that of the chemical group (87.46%). Survival analysis showed that median clinical recovery time was 3 days in YHGMS combined group, 4 days in only using YHGMS group and 4 days in chemical group. The time to return normal temperature in the 3 groups was 1 day, 1 day and 2 days, respectively. The difference between groups was statistically significant (*P* < 0.05). The resolution rate of each symptom in YHGMS combined group was higher than that in chemical group. Resolution rate of purulent rhinorrhea at all time points, resolution rate of sneezing, expectoration, dysphoria and thirst at day 5 and day 7, of sweat at day 3, of stuffy nose, wind aversion, dark urine at day 5, and of cough at day 7 were significantly higher in YHGMS only group. Differences between groups were statistically significant (*P* < 0.0167). In addition, we also conducted subgroup analysis of YHGMS with compound preparations, antibiotics/antiviral drugs, antipyretic analgesics, and other chemical medicines respectively, and the results were basically consistent with this subgroup analysis (Supplementary Tables S3–S8).

#### 3.4.2 Effect of YHGMS applied at different acupoints and chemical medicine

The children who were administered YHGMS at a single acupoint were divided into two groups based on the acupoints to which they were applied: Shenque group and Dazhui group and then compared with children with chemical group. On day 3 and day 5, recovery rate of Dazhui group was the highest (72.41% and 93.10%), followed by Shenque group (53.56% and 81.23%), and the lowest in chemical group (36.09% and 68.81%). On day 7, the recovery rates of groups treated with YHGMS reached about 95%, which was higher than 87.46% in chemical group. There was no statistical difference in median recovery time between Shenque group and Dazhui group, but both were shorter than chemical group. The result for time to return normal temperature was consistent with that of recovery time.

Furthermore, we conducted similar subgroup analysis in children treated with YHGMS only and YHGMS combination group, and the results were found to be largely consistent (Supplementary Tables S9–S11).

#### 3.4.3 Other subgroup analysis

Table 5 presents subgroup analysis according to fever, age group and duration of disease. Recovery rate of children under 2 years old is higher than that of other age groups, and the recovery rate of group in above 6 years old (including), 2–4 years old and 4–6 years old decreased successively. As for the disease course, the resolution rate was highest at each time point in patients with a disease course of 0–24 h, followed by 24–48 h; those with a course exceeding 48 h consistently demonstrated the least resolution rates. According to the grouping based on fever, the recovery rate of febrile group was higher than that of non-febrile group. Regardless of fever, the recovery time was shorter and the recovery rate was higher in YHGMS group than that of those who were not treated with YHGMS (*P* < 0.05).

**TABLE 5:** Univariate analysis in effect of characteristics on recovery rate of upper respiratory tract infection (%).

Factors	Day 3		Day 5		Day 7	
	Exposed (n = 663)	Non-exposed (n = 327)	Exposed (n = 663)	Non-exposed (n = 327)	Exposed (n = 663)	Non-exposed (n = 327)
Fever						
Yes	58.66	39.81	85.93	74.27	95.67	87.86
No	38.81	29.75	69.65	59.50	92.54	86.78
Age group						
0 years∼	60.00	36.59	88.50	69.51	95.50	89.02
2 years∼	51.65	33.33	77.47	61.54	92.86	82.05
4 years∼	42.96	32.89	77.78	61.84	94.07	80.26
6 years∼	52.74	40.66	78.08	80.22	96.58	96.70
Duration						
0 h∼	63.57	41.30	85.27	69.57	95.35	76.09
24 h∼	54.96	33.56	82.82	69.86	93.13	90.14
48 h∼	45.22	37.04	77.21	67.41	95.96	88.15

### 3.5 Safety

A total of 19 adverse events occurred in 18 children in the YHGMS exposed group and the incidence was 2.71% (event incidence was 2.85%), and no serious adverse events were reported.

Among the 19 events, 10 were judged to be possibly related to YHGMS, and the incidence of adverse reactions of YHGMS was 1.51%. All the adverse events were characterized by redness, swelling and itching of the skin at application site and no systemic reactions occurred. Local adverse reactions resolved spontaneously after discontinuation or dose reduction, without treatment. (see Supplementary Table S12 for details).

## 4 Discussion

In this large, prospective, real-world cohort study of 990 children with AURTI of the wind-heat pattern, YHGMS was associated with better clinical outcomes and a favorable safety profile compared with conventional care. Although there was a slight age difference between groups at baseline (*P* = 0.0280), PSM successfully balanced this and other potential confounders, as shown in Table 1. Our findings are consistent with previous meta-analyses showing that TCM can shorten fever duration and improve symptoms in pediatric AURTI (Qiu et al., 2014; Ouyang et al., 2022). Globally, complementary and alternative medicine approaches, including TCM, are widely used for pediatric respiratory infections (Hawke et al., 2022). However, the evidence base for such interventions remains heterogeneous and evolving.

The 1-day reduction in recovery time and fever duration is modest but may still be clinically meaningful for children and their caregivers. It translates to earlier return to normal activities and reduced healthcare burden, and may help alleviate parental anxiety (Thompson et al., 2013; Yan et al., 2022). Nevertheless, given the self-limiting nature of AURTI, the clinical significance of a single-day reduction should not be overstated. Instead, the primary value of YHGMS may lie in two aspects. First, the favorable safety profile, with only 1.51% mild, self-limiting local skin reactions, supports the use of YHGMS as a well-tolerated option for pediatric AURTI. Moreover, the observed lower use of antibiotics, antivirals, and other chemical medicines in the YHGMS group aligns with the global antimicrobial stewardship agenda (World Health Organization, 2015; Centers for Disease Control and Prevention, 2026). This consistency is further supported by recent international recommendations that endorse non-antibiotic strategies for uncomplicated AURTI (Zanichelli et al., 2023). A recent Italian study reported that nearly 25% of children received unnecessary antibiotics for viral upper respiratory tract infections, highlighting the widespread nature of this issue (Bianco et al., 2022). By offering a non-oral, well-tolerated option, YHGMS may help reduce unnecessary antibiotic prescribing for viral AURTI, a key driver of antimicrobial resistance.

Although this multicenter prospective cohort study enhances the reliability and generalizability of the results, there are still some limitations. First, the non-randomized allocation carries a risk of selection bias and confounding by indication. Although PSM adjusted for measured covariates, unmeasured confounders (e.g., parental health beliefs, illness severity perception) may still have influenced treatment choice and outcomes. Second, blinding of patients, caregivers, and physicians was not feasible due to the distinctive odor and appearance of the patch, which may have introduced performance bias, and a placebo effect cannot be excluded. Third, subtle differences among centers in case inclusion and treatment decisions may affect consistency. We used a 2:1 enrollment quota per center to ensure balanced group distribution within each site, reducing potential confounding by center, and provided standardized training before the study. Fourth, outcome assessment relied in part on parent-completed diary cards, which are inherently subjective and may introduce reporting bias, despite standardized training. Finally, the subgroup analyses were exploratory; the multiple comparisons increase the risk of type I error, and these results should be interpreted cautiously.

## 5 Conclusion

YHGMS was associated with faster clinical recovery and symptom resolution in children with AURTI (wind-heat pattern), without significant safety concerns. As monotherapy, YHGMS showed better outcomes than chemical medicine alone; in combination, it also accelerated resolution of clinical symptoms and recovery. These findings support YHGMS as a viable non-oral option for the treatment of AURTI in children. However, due to the non-randomized design, causality cannot be inferred; randomized controlled trials are needed to confirm these associations.

## Data Availability

The raw data supporting the conclusions of this article will be made available by the authors, without undue reservation.
