# Supplementary material for: Treatment of children with acute upper respiratory tract infection (wind-heat pattern) with Yin Hu Gan Mao San: a prospective real-world cohort study
Source: Front Pharmacol. 2026 Jul 2;17:1838314. doi: 10.3389/fphar.2026.1838314 (PMC13372784; doi:10.3389/fphar.2026.1838314)
Supplement: Supplementary file 1 [file Table1.DOCX]

| Table S1 Criteria for Syndrome Differentiation in Traditional Chinese Medicine (Wind-Heat Pattern) | |
| --- | --- |
| Main Symptoms | 1. Fever; ②Stuffy nose; ③Purulent rhinorrhea; ④Sneeze; ⑤Sore throat; ⑥Cough. |
| Secondary Symptoms | ①Wind aversion; ②Sweat; ③Expectoration; ④Flush face; ⑤Dysphoria; ⑥Headache; ⑦Thirst; ⑧Dark urine |
| Tongue, Pulse, and Finger Vein | Red tongue with thin yellow coating, floating and rapid pulse, floating purple finger vein. |
| Diagnosis can be made with at least 3 main symptoms and at least 4 secondary symptoms, with reference to tongue, pulse, and finger vein findings. | |

| Table S2. Resolution rate of each symptom at different time nodes (%)- Detail | | | | |
| --- | --- | --- | --- | --- |
| Symptoms | Exposed group (n=663) | Non-exposed group (n=327) | Statistic | *P* value |
| **Fever-baseline** |  |  |  |  |
| No n(%) | 205(30.92) | 121(37.00) | *Z*=0.3237 | 0.7462 |
| Mild n(%) | 62(9.35) | 20(6.12) |  |  |
| Moderate n(%) | 238(35.90) | 81(24.77) |  |  |
| Severe n(%) | 158(23.83) | 105(32.11) |  |  |
| **Resolution rate of fever at day 3** | |  |  |  |
| Resolved, n(%) | 394(86.03) | 152(73.79) | *χ^2^*=14.5671 | **0.0001** |
| Not resolved, n(%) | 64(13.97) | 54(26.21) |  |  |
| **Resolution rate of fever at day 5** | |  |  |  |
| Resolved, n(%) | 436(95.20) | 196(95.15) | *χ^2^*=0.0008 | 0.9774 |
| Not resolved, n(%) | 22(4.80) | 10(4.85) |  |  |
| **Resolution rate of fever at day 7** | |  |  |  |
| Resolved, n(%) | 457(99.78) | 204(99.03) | Fisher | 0.2287 |
| Not resolved, n(%) | 1(0.22) | 2(0.97) |  |  |
| **Stuffy nose-baseline** |  |  |  |  |
| No, n(%) | 150(22.62) | 73(22.32) | *Z*=1.7190 | 0.0856 |
| Mild, n(%) | 315(47.51) | 131(40.06) |  |  |
| Moderate, n(%) | 175(26.40) | 108(33.03) |  |  |
| Severe, n(%) | 23(3.47) | 15(4.59) |  |  |
| **Resolution rate of stuffy nose fever at day 3** | |  |  |  |
| Resolved, n(%) | 336(65.50) | 133(52.36) | *χ^2^*=12.3370 | **0.0004** |
| Not resolved, n(%) | 177(34.50) | 121(47.64) |  |  |
| **Resolution rate of stuffy nose at day 5** | |  |  |  |
| Resolved, n(%) | 439(85.58) | 183(72.05) | *χ^2^*=20.2788 | **<.0001** |
| Not resolved, n(%) | 74(14.42) | 71(27.95) |  |  |
| **Resolution rate of stuffy nose at day 7** | |  |  |  |
| Resolved, n(%) | 467(91.03) | 215(84.65) | *χ^2^*=7.0340 | **0.0080** |
| Not resolved, n(%) | 46(8.97) | 39(15.35) |  |  |
| **Purulent rhinorrhea- baseline** |  |  |  |  |
| No, n(%) | 125(18.85) | 62(18.96) | *Z*=1.5663 | 0.1173 |
| Mild, n(%) | 280(42.23) | 116(35.47) |  |  |
| Moderate, n(%) | 231(34.84) | 130(39.76) |  |  |
| Severe, n(%) | 27(4.07) | 19(5.81) |  |  |
| **Resolution rate of purulent rhinorrhea at day 3** | |  |  |  |
| Resolved, n(%) | 342(63.57) | 129(48.68) | *χ^2^*=16.2310 | **<.0001** |
| Not resolved, n(%) | 196(36.43) | 136(51.32) |  |  |
| **Resolution rate of purulent rhinorrhea at day 5** | |  |  |  |
| Resolved, n(%) | 431(80.11) | 175(66.04) | *χ^2^*=18.9945 | **<.0001** |
| Not resolved, n(%) | 107(19.89) | 90(33.96) |  |  |
| **Resolution rate of purulent rhinorrhea at day 7** | |  |  |  |
| Resolved, n(%) | 480(89.22) | 210(79.25) | *χ^2^*=14.6069 | **0.0001** |
| Not resolved, n(%) | 58(10.78) | 55(20.75) |  |  |
| **Sneeze- baseline** |  |  |  |  |
| No, n(%) | 220(33.18) | 111(33.94) | *Z*=0.4623 | 0.6439 |
| Mild, n(%) | 342(51.58) | 155(47.40) |  |  |
| Moderate, n(%) | 93(14.03) | 56(17.13) |  |  |
| Severe, n(%) | 8(1.21) | 5(1.53) |  |  |
| **Resolution rate of sneeze at day 3** | |  |  |  |
| Resolved, n(%) | 323(72.91) | 127(58.80) | *χ^2^*=13.3594 | **0.0003** |
| Not resolved, n(%) | 120(27.09) | 89(41.20) |  |  |
| **Resolution rate of sneeze at day 5** | |  |  |  |
| Resolved, n(%) | 382(86.23) | 159(73.61) | *χ^2^*=15.7298 | **<.0001** |
| Not resolved, n(%) | 61(13.77) | 57(26.39) |  |  |
| **Resolution rate of sneeze at day 7** | |  |  |  |
| Resolved, n(%) | 420(94.81) | 176(81.48) | *χ^2^*=29.8262 | **<.0001** |
| Not resolved, n(%) | 23(5.19) | 40(18.52) |  |  |
| **Sore throat- baseline** |  |  |  |  |
| No, n(%) | 104(15.69) | 44(13.46) | *Z*=2.0215 | **0.0432** |
| Mild, n(%) | 363(54.75) | 165(50.46) |  |  |
| Moderate, n(%) | 192(28.96) | 115(35.17) |  |  |
| Severe, n(%) | 4(0.60) | 3(0.92) |  |  |
| **Resolution rate of sore throat at day 3** | |  |  |  |
| Resolved, n(%) | 381(68.16) | 171(60.42) | *χ^2^*=4.9764 | **0.0257** |
| Not resolved, n(%) | 178(31.84) | 112(39.58) |  |  |
| **Resolution rate of sore throat at day 5** | |  |  |  |
| Resolved, n(%) | 467(83.54) | 216(76.33) | *χ^2^*=6.3885 | **0.0115** |
| Not resolved, n(%) | 92(16.46) | 67(23.67) |  |  |
| **Resolution rate of sore throat at day 7** | |  |  |  |
| Resolved, n(%) | 492(88.01) | 232(81.98) | *χ^2^*=5.6796 | **0.0172** |
| Not resolved, n(%) | 67(11.99) | 51(18.02) |  |  |
| **Cough- baseline** |  |  |  |  |
| No, n(%) | 211(31.83) | 89(27.22) | *Z*=2.9828 | **0.0029** |
| Mild, n(%) | 270(40.72) | 111(33.94) |  |  |
| Moderate, n(%) | 174(26.24) | 123(37.61) |  |  |
| Severe, n(%) | 8(1.21) | 4(1.22) |  |  |
| **Resolution rate of cough at day 3** | |  |  |  |
| Resolved, n(%) | 244(53.98) | 103(43.28) | *χ^2^*=7.1468 | **0.0075** |
| Not resolved, n(%) | 208(46.02) | 135(56.72) |  |  |
| **Resolution rate of cough at day 5** | |  |  |  |
| Resolved, n(%) | 333(73.67) | 145(60.92) | *χ^2^*=11.9041 | **0.0006** |
| Not resolved, n(%) | 119(26.33) | 93(39.08) |  |  |
| **Resolution rate of cough at day 7** | |  |  |  |
| Resolved, n(%) | 370(81.86) | 167(70.17) | *χ^2^*=12.3468 | **0.0004** |
| Not resolved, n(%) | 82(18.14) | 71(29.83) |  |  |
| **Wind aversion- baseline** |  |  |  |  |
| No, n(%) | 388(58.52) | 190(58.10) | *χ^2^*=0.0157 | 0.9002 |
| Yes, n(%) | 275(41.48) | 137(41.90) |  |  |
| **Resolution rate of wind aversion at day 3** | |  |  |  |
| Resolved n(%) | 186(67.64) | 75(54.74) | *χ^2^*=6.5458 | **0.0105** |
| Not resolved n(%) | 89(32.36) | 62(45.26) |  |  |
| **Resolution rate of wind aversion at day 5** | |  |  |  |
| Resolved n(%) | 251(91.27) | 101(73.72) | *χ^2^*=22.6368 | **<.0001** |
| Not resolved n(%) | 24(8.73) | 36(26.28) |  |  |
| **Resolution rate of wind aversion at day 7** | |  |  |  |
| Resolved n(%) | 270(98.18) | 126(91.97) | *χ^2^*=9.4506 | **0.0021** |
| Not resolved n(%) | 5(1.82) | 11(8.03) |  |  |
| **Sweat-baseline** |  |  |  |  |
| No, n(%) | 146(22.02) | 63(19.27) | *χ^2^*=0.9981 | 0.3178 |
| Yes, n(%) | 517(77.98) | 264(80.73) |  |  |
| **Resolution rate of sweat at day 3** | |  |  |  |
| Resolved n(%) | 309(59.77) | 113(42.80) | *χ^2^*=20.2507 | **<.0001** |
| Not resolved n(%) | 208(40.23) | 151(57.20) |  |  |
| **Resolution rate of sweat at day 5** | |  |  |  |
| Resolved n(%) | 426(82.40) | 183(69.32) | *χ^2^*=17.4114 | **<.0001** |
| Not resolved n(%) | 91(17.60) | 81(30.68) |  |  |
| **Resolution rate of sweat at day 7** | |  |  |  |
| Resolved n(%) | 471(91.10) | 212(80.30) | *χ^2^*=18.5740 | **<.0001** |
| Not resolved n(%) | 46(8.90) | 52(19.70) |  |  |
| **Expectoration- baseline** |  |  |  |  |
| No, n(%) | 341(51.43) | 155(47.40) | *χ^2^*=1.4243 | 0.2327 |
| Yes, n(%) | 322(48.57) | 172(52.60) |  |  |
| **Resolution rate of expectoration at day 3** | |  |  |  |
| Resolved n(%) | 193(59.94) | 80(46.51) | *χ^2^*=8.1746 | **0.0043** |
| Not resolved n(%) | 129(40.06) | 92(53.49) |  |  |
| **Resolution rate of expectoration at day 5** | |  |  |  |
| Resolved n(%) | 234(72.67) | 99(57.56) | *χ^2^*=11.6553 | **0.0006** |
| Not resolved n(%) | 88(27.33) | 73(42.44) |  |  |
| **Resolution rate of expectoration at day 7** | |  |  |  |
| Resolved n(%) | 277(86.02) | 124(72.09) | *χ^2^*=14.2397 | **0.0002** |
| Not resolved n(%) | 45(13.98) | 48(27.91) |  |  |
| **Flush face- baseline** |  |  |  |  |
| No, n(%) | 247(37.25) | 128(39.14) | *χ^2^*=0.3320 | 0.5645 |
| Yes, n(%) | 416(62.75) | 199(60.86) |  |  |
| **Resolution rate of flush face at day 3** | |  |  |  |
| Resolved n(%) | 294(70.67) | 112(56.28) | *χ^2^*=12.4272 | **0.0004** |
| Not resolved n(%) | 122(29.33) | 87(43.72) |  |  |
| **Resolution rate of flush face at day 5** | |  |  |  |
| Resolved n(%) | 369(88.70) | 152(76.38) | *χ^2^*=15.7790 | **<.0001** |
| Not resolved n(%) | 47(11.30) | 47(23.62) |  |  |
| **Resolution rate of flush face at day 7** | |  |  |  |
| Resolved n(%) | 399(95.91) | 168(84.42) | *χ^2^*=24.7024 | **<.0001** |
| Not resolved n(%) | 17(4.09) | 31(15.58) |  |  |
| **Dysphoria- baseline** | |  |  |  |
| No, n(%) | 324(48.87) | 191(58.41) | *χ^2^*=7.9870 | **0.0047** |
| Yes, n(%) | 339(51.13) | 136(41.59) |  |  |
| **Resolution rate of dysphoria at day 3** | |  |  |  |
| Resolved n(%) | 197(58.11) | 55(40.44) | *χ^2^*=12.1687 | **0.0005** |
| Not resolved n(%) | 142(41.89) | 81(59.56) |  |  |
| **Resolution rate of dysphoria at day 5** | |  |  |  |
| Resolved n(%) | 282(83.19) | 90(66.18) | *χ^2^*=16.5359 | **<.0001** |
| Not resolved n(%) | 57(16.81) | 46(33.82) |  |  |
| **Resolution rate of dysphoria at day 7** | |  |  |  |
| Resolved n(%) | 322(94.99) | 108(79.41) | *χ^2^*=27.4488 | **<.0001** |
| Not resolved n(%) | 17(5.01) | 28(20.59) |  |  |
| **Headache- baseline** |  |  |  |  |
| No, n(%) | 526(79.34) | 232(70.95) | *χ^2^*=8.5880 | **0.0034** |
| Yes, n(%) | 137(20.66) | 95(29.05) |  |  |
| **Resolution rate of headache at day 3** | |  |  |  |
| Resolved n(%) | 98(71.53) | 52(54.74) | *χ^2^*=6.9253 | **0.0085** |
| Not resolved n(%) | 39(28.47) | 43(45.26) |  |  |
| **Resolution rate of headache at day 5** | |  |  |  |
| Resolved n(%) | 124(90.51) | 77(81.05) | *χ^2^*=4.3351 | **0.0373** |
| Not resolved n(%) | 13(9.49) | 18(18.95) |  |  |
| **Resolution rate of headache at day 7** | |  |  |  |
| Resolved n(%) | 133(97.08) | 86(90.53) | *χ^2^*=4.5557 | **0.0328** |
| Not resolved n(%) | 4(2.92) | 9(9.47) |  |  |
| **Thirst- baseline** |  |  |  |  |
| No, n(%) | 259(39.06) | 122(37.31) | *χ^2^*=0.2852 | 0.5933 |
| Yes, n(%) | 404(60.94) | 205(62.69) |  |  |
| **Resolution rate of thirst at day 3** |  |  |  |  |
| Resolved n(%) | 210(51.98) | 71(34.63) | *χ^2^*=16.4655 | **<.0001** |
| Not resolved n(%) | 194(48.02) | 134(65.37) |  |  |
| **Resolution rate of thirst at day 5** |  |  |  |  |
| Resolved n(%) | 322(79.70) | 124(60.49) | *χ^2^*=25.6164 | **<.0001** |
| Not resolved n(%) | 82(20.30) | 81(39.51) |  |  |
| **Resolution rate of thirst at day 7** |  |  |  |  |
| Resolved n(%) | 367(90.84) | 153(74.63) | *χ^2^*=28.6278 | **<.0001** |
| Not resolved n(%) | 37(9.16) | 52(25.37) |  |  |
| **Dark urine- baseline** |  |  |  |  |
| No, n(%) | 107(16.14) | 64(19.57) | *χ^2^*=1.8063 | 0.1790 |
| Yes, n(%) | 556(83.86) | 263(80.43) |  |  |
| **Resolution rate of dark urine at day 3** | |  |  |  |
| Resolved n(%) | 219(39.39) | 85(32.32) | *χ^2^*=3.8226 | 0.0506 |
| Not resolved n(%) | 337(60.61) | 178(67.68) |  |  |
| **Resolution rate of dark urine at day 5** | |  |  |  |
| Resolved n(%) | 370(66.55) | 136(51.71) | *χ^2^*=16.6432 | **<.0001** |
| Not resolved n(%) | 186(33.45) | 127(48.29) |  |  |
| **Resolution rate of dark urine at day 7** | |  |  |  |
| Resolved n(%) | 438(78.78) | 179(68.06) | *χ^2^*=11.0346 | **0.0009** |
| Not resolved n(%) | 118(21.22) | 84(31.94) |  |  |

| Table S3. Analysis of clinical recovery of upper respiratory tract infection in subgroup analysis-1 (%) | | | | | |
| --- | --- | --- | --- | --- | --- |
|  | Yin Hu Gan Mao San | | Chemical group  (n=327) | Statistic | *P* value |
|  | Only using YHGMS group (n=319) | YHGMS combined group (n=344) |  |  |  |
| **Recovery rate at day 3** | 152(47.65) | 197(57.27) | 118(36.09) | *χ^2^*=30.2260 | **<.0001** |
| Chemical group *vs* only using YHGMS group | | |  | *χ^2^*=8.8753 | **0.0029** |
| Chemical group *vs* YHGMS combined group | | |  | *χ^2^*=30.1991 | **<.0001** |
| Only using YHGMS group *vs* YHGMS combined group | | |  | *χ^2^*=6.1422 | **0.0132** |
| **Recovery rate at day 5** | 245(76.80) | 292(84.88) | 225(68.81) | *χ^2^*=24.4497 | **<.0001** |
| Chemical group *vs* only using YHGMS group | | |  | *χ^2^*=5.2073 | 0.0225 |
| Chemical group *vs* YHGMS combined group | | |  | *χ^2^*=24.5016 | **<.0001** |
| Only using YHGMS group *vs* YHGMS combined group | | |  | *χ^2^*=7.0222 | **0.0081** |
| **Recovery rate at day 7** | 308(96.55) | 320(93.02) | 286(87.46) | *χ^2^*=19.1897 | **<.0001** |
| Chemical group *vs* only using YHGMS group | | |  | *χ^2^*=18.0262 | **<.0001** |
| Chemical group *vs* YHGMS combined group | | |  | *χ^2^*=5.9268 | **0.0149** |
| Only using YHGMS group *vs* YHGMS combined group | | |  | *χ^2^*=4.1210 | 0.0424 |
| **Recovery time (day)** | 4.0(2.0,5.0) | 3.0(3.0,5.0) | 4.0(3.0,6.0) | 23.5107 | <.0001 |
| Chemical group *vs* only using YHGMS group | | |  | 8.5662 | 0.0034 |
| Chemical group *vs* YHGMS combined group | | |  | 23.9418 | <.0001 |
| Only using YHGMS group *vs* YHGMS combined group | | |  | 2.6183 | 0.1056 |
| **Time to return normal temperature (day)** | 1.0(1.0,3.0) | 1.0(1.0,2.0) | 2.0(2.0,4.0) | 30.0801 | <.0001 |
| Chemical group *vs* only using YHGMS group | | |  | 12.5744 | 0.0004 |
| Chemical group *vs* YHGMS combined group | | |  | 32.0093 | <.0001 |
| Only using YHGMS group *vs* YHGMS combined group | | |  | 1.7163 | 0.1902 |

| Table S4. Resolution rate of each symptom at different time nodes in subgroup analysis -1 (%) | | | | | | | | | | | |
| --- | --- | --- | --- | --- | --- | --- | --- | --- | --- | --- | --- |
| Symptom | Day 3 | | |  | Day 5 | | |  | Day 7 | | |
|  | Only using YHGMS group | YHGMS combined group | Chemical group |  | Only using YHGMS group | YHGMS combined group | Chemical group |  | Only using YHGMS group | YHGMS combined group | Chemical group |
| Fever | 81.67 | 88.85 | 73.79 |  | 93.89 | 96.04 | 95.15 |  | 99.44 | 100.00 | 99.03 |
| Stuffy nose | 62.26 | 68.75 | 52.36 |  | 82.88 | 88.28 | 72.05 |  | 89.49 | 92.58 | 84.65 |
| Purulent rhinorrhea | 63.57 | 63.57 | 48.68 |  | 77.91 | 82.14 | 66.04 |  | 89.92 | 88.57 | 79.25 |
| Sneeze | 69.41 | 76.34 | 58.80 |  | 83.56 | 88.84 | 73.61 |  | 95.89 | 93.75 | 81.48 |
| Sore throat | 64.37 | 71.48 | 60.42 |  | 81.23 | 85.57 | 76.33 |  | 87.36 | 88.59 | 81.98 |
| Cough | 51.23 | 56.22 | 43.28 |  | 70.94 | 75.90 | 60.92 |  | 83.74 | 80.32 | 70.17 |
| Wind aversion | 63.16 | 70.81 | 54.74 |  | 88.60 | 93.17 | 73.72 |  | 97.37 | 98.76 | 91.97 |
| Sweat | 54.63 | 63.79 | 42.80 |  | 78.75 | 85.17 | 69.32 |  | 86.34 | 94.83 | 80.30 |
| Expectoration | 58.22 | 61.36 | 46.51 |  | 71.23 | 73.86 | 57.56 |  | 84.93 | 86.93 | 72.09 |
| Flush face | 65.17 | 74.79 | 56.28 |  | 83.71 | 92.44 | 76.38 |  | 91.57 | 99.16 | 84.42 |
| Dysphoria | 53.09 | 62.71 | 40.44 |  | 82.10 | 84.18 | 66.18 |  | 92.59 | 97.18 | 79.41 |
| Headache | 69.12 | 73.91 | 54.74 |  | 92.65 | 88.41 | 81.05 |  | 95.59 | 98.55 | 90.53 |
| Thirst | 45.41 | 58.88 | 34.63 |  | 73.43 | 86.29 | 60.49 |  | 86.96 | 94.92 | 74.63 |
| Dark urine | 35.45 | 43.06 | 32.32 |  | 62.69 | 70.14 | 51.71 |  | 75.75 | 81.60 | 68.06 |

| Table S5. Analysis of clinical recovery of upper respiratory tract infection in subgroup analysis-2 (%) | | | | | |
| --- | --- | --- | --- | --- | --- |
|  | Yin Hu Gan Mao San | | Compound group  (n=106) | Statistic | *P* value |
|  | Only using YHGMS group  (n=319) | YHGMS combined group (n=143) |  |  |  |
| **Recovery rate at day 3** | 152(47.65) | 81(56.64) | 48(45.28) | *χ^2^*=4.1103 | 0.1281 |
| **Recovery rate at day 5** | 245(76.80) | 123(86.01) | 85(80.19) | *χ^2^*=5.2038 | 0.0741 |
| **Recovery rate at day 7** | 308(96.55) | 134(93.71) | 92(86.79) | *χ^2^*=13.4976 | **0.0012** |
| Compound group *vs* only using YHGMS group | | |  | *χ^2^*=13.6874 | **0.0002** |
| Compound group *vs* YHGMS combined group | | |  | *χ^2^*=3.4709 | 0.0625 |
| Only using YHGMS group *vs* YHGMS combined group | | |  | *χ^2^*=1.9302 | 0.1647 |
| **Recovery time (day)** | 4.0(2.0,5.0) | 3.0(3.0,5.0) | 4.0(3.0,5.0) | 2.1110 | 0.3480 |
| **Time to return normal temperature (day)** | 1.0(1.0,3.0) | 1.5(1.0,3.0) | 2.0(1.0,3.0) | 1.2560 | 0.5337 |

| Table S6. Analysis of clinical recovery of upper respiratory tract infection in subgroup analysis-3 (%) | | | | | |
| --- | --- | --- | --- | --- | --- |
|  | Yin Hu Gan Mao San | | Antibiotics/antivirus group (n=87) | Statistic | *P* value |
|  | Only using YHGMS group  (n=319) | YHGMS combined group (n=80) |  |  |  |
| **Recovery rate at day 3** | 152(47.65) | 50(62.50) | 31(35.63) | *χ^2^*=12.0867 | **0.0024** |
| Antibiotics/antivirus group *vs* only using YHGMS group | | |  | *χ^2^*=3.9871 | 0.0459 |
| Antibiotics/antivirus group *vs* YHGMS combined group | | |  | *χ^2^*=12.0450 | **0.0005** |
| Only using YHGMS group *vs* YHGMS combined group | | |  | *χ^2^*=5.6436 | 0.0175 |
| **Recovery rate at day 5** | 245(76.80) | 68(85.00) | 69(79.31) | *χ^2^*=2.5870 | 0.2743 |
| **Recovery rate at day 7** | 308(96.55) | 71(88.75) | 80(91.95) | Fisher | **0.0114** |
| Antibiotics/antivirus group *vs* only using YHGMS group | | |  | Fisher | 0.0781 |
| Antibiotics/antivirus group *vs* YHGMS combined group | | |  | *χ^2^*=0.4939 | 0.4822 |
| Only using YHGMS group *vs* YHGMS combined group | | |  | Fisher | **0.0084** |
| **Recovery time (day)** | 4.0(2.0,5.0) | 3.0(3.0,5.0) | 4.0(3.0,5.0) | 0.8081 | 0.6676 |
| **Time to return normal temperature (day)** | 1.0(1.0,3.0) | 1.0(1.0,2.0) | 3.00(2.0,4.0) | 21.0701 | <.0001 |
| Antibiotics/antivirus group *vs* only using YHGMS group | | |  | 9.5289 | 0.0020 |
| Antibiotics/antivirus group *vs* YHGMS combined group | | |  | 27.3659 | <.0001 |
| Only using YHGMS group *vs* YHGMS combined group | | |  | 4.8767 | 0.0272 |

| Table S7. Analysis of clinical recovery of upper respiratory tract infection in subgroup analysis-4 (%) | | | | | | |
| --- | --- | --- | --- | --- | --- | --- |
|  | Yin Hu Gan Mao San | | | Antipyretic group (n=142) | Statistic | *P* value |
|  | Only using YHGMS group  (n=319) | YHGMS combined group (n=131) | |  |  |  |
| **Recovery rate at day 3** | 152(47.65) | 88(70.97) | | 25(34.72) | *χ^2^*=28.8216 | **<.0001** |
| Antipyretic group *vs* only using YHGMS group | | | |  | *χ^2^*=3.9617 | 0.0465 |
| Antipyretic group *vs* YHGMS combined group | | | |  | *χ^2^*=24.5111 | **<.0001** |
| Only using YHGMS group *vs* YHGMS combined group | | | |  | *χ^2^*=19.5579 | **<.0001** |
| **Recovery rate at day 5** | 245(76.80) | 111(89.52) | | 43(59.72) | *χ^2^*=23.3883 | **<.0001** |
| Antipyretic group *vs* only using YHGMS group | | | |  | *χ^2^*=8.8320 | **0.0030** |
| Antipyretic group *vs* YHGMS combined group | | | |  | *χ^2^*=24.0157 | **<.0001** |
| Only using YHGMS group *vs* YHGMS combined group | | | |  | *χ^2^*=9.1450 | **0.0025** |
| **Recovery rate at day 7** | 308(96.55) | 121(97.58) | | 60(83.33) | Fisher | **0.0001** |
| Antipyretic group *vs* only using YHGMS group | | | |  | Fisher | **0.0002** |
| Antipyretic group *vs* YHGMS combined group | | | |  | *χ^2^*=13.0830 | **0.0003** |
| Only using YHGMS group *vs* YHGMS combined group | | | |  | Fisher | 0.7659 |
| **Recovery time (day)** | 4.0(2.0,5.0) | | 3.0(2.0,4.0) | 5.0(3.0,6.0) | 36.4653 | <.0001 |
| Antipyretic group *vs* only using YHGMS group | | | |  | 8.1552 | 0.0043 |
| Antipyretic group *vs* YHGMS combined group | | | |  | 32.3795 | <.0001 |
| Only using YHGMS group *vs* YHGMS combined group | | | |  | 20.7494 | <.0001 |
| **Time to return normal temperature (day)** | 1.0(1.0,3.0) | | 2.0(1.0,2.0) | 3.0(2.0,4.0) | 17.2705 | 0.0002 |
| Antipyretic group *vs* only using YHGMS group | | | |  | 9.2933 | 0.0023 |
| Antipyretic group *vs* YHGMS combined group | | | |  | 22.0717 | <.0001 |
| Only using YHGMS group *vs* YHGMS combined group | | | |  | 1.2361 | 0.2662 |

| Table S8. Analysis of clinical recovery of upper respiratory tract infection in subgroup analysis-5 (%) | | | | | |
| --- | --- | --- | --- | --- | --- |
|  | Yin Hu Gan Mao San | | Other chemical group (n=142) | Statistic | *P* value |
|  | Only using YHGMS group  (n=319) | YHGMS combined group (n=131) |  |  |  |
| **Recovery rate at day 3** | 152(47.65) | 73(55.73) | 41(28.87) | *χ^2^*=21.9191 | **<.0001** |
| Other chemical group *vs* only using YHGMS group | | |  | *χ^2^*=14.2324 | **0.0002** |
| Other chemical group *vs* YHGMS combined group | | |  | *χ^2^*=20.2010 | **<.0001** |
| Only using YHGMS group *vs* YHGMS combined group | | |  | *χ^2^*=2.4229 | 0.1196 |
| **Recovery rate at day 5** | 245(76.80) | 110(83.97) | 76(53.52) | *χ^2^*=37.4911 | **<.0001** |
| Other chemical group *vs* only using YHGMS group | | |  | *χ^2^*=23.3786 | **<.0001** |
| Other chemical group *vs* YHGMS combined group | | |  | *χ^2^*=8.2063 | **0.0042** |
| Only using YHGMS group *vs* YHGMS combined group | | |  | *χ^2^*=0.8686 | 0.3513 |
| **Recovery rate at day 7** | 308(96.55) | 124(94.66) | 119(83.80) | *χ^2^*=25.4293 | **<.0001** |
| Other chemical group *vs* only using YHGMS group | | |  | *χ^2^*=24.2504 | **<.0001** |
| Other chemical group *vs* YHGMS combined group | | |  | *χ^2^*=7.5730 | **0.0059** |
| Only using YHGMS group *vs* YHGMS combined group | | |  | Fisher | 0.3635 |
| **Recovery time (day)** | 4.0(2.0,5.0) | 3.0(2.0,4.0) | 5.0(3.0,7.0) | 34.6945 | <.0001 |
| Other chemical group *vs* only using YHGMS group | | |  | 23.1942 | <.0001 |
| Other chemical group *vs* YHGMS combined group | | |  | 32.7697 | <.0001 |
| Only using YHGMS group *vs* YHGMS combined group | | |  | 3.3387 | 0.0677 |
| **Time to return normal temperature (day)** | 1.0(1.0,3.0) | 2.0(1.0,2.0) | 3.0(2.0,4.0) | 17.2705 | 0.0002 |
| Other chemical group *vs* only using YHGMS group | | |  | 9.2488 | 0.0024 |
| Other chemical group *vs* YHGMS combined group | | |  | 25.5285 | <.0001 |
| Only using YHGMS group *vs* YHGMS combined group | | |  | 2.2176 | 0.1364 |

| Table S9. Analysis of clinical recovery of upper respiratory tract infection in subgroup analysis-6 (%) | | | | | |
| --- | --- | --- | --- | --- | --- |
|  | Yin Hu Gan Mao San | | Chemical group (n=327) | Statistic | *P* value |
|  | Shenque group (n=506) | Dazhui group (n=29) |  |  |  |
| **Recovery rate at day 3** | 271(53.56) | 21(72.41) | 118(36.09) | *χ^2^*=31.7420 | **<.0001** |
| Chemical group *vs* Shenque group | | |  | *χ^2^*=24.3602 | **<.0001** |
| Chemical group *vs* Dazhui group | | |  | *χ^2^*=14.7709 | **<.0001** |
| Shenque group *vs* Dazhui group | | |  | *χ^2^*=3.9340 | 0.0473 |
| **Recovery rate at day 5** | 411(81.23) | 27(93.10) | 225(68.81) | *χ^2^*=21.6801 | **<.0001** |
| Chemical group *vs* Shenque group | | |  | *χ^2^*=16.9637 | **<.0001** |
| Chemical group *vs* Dazhui group | | |  | *χ^2^*=7.6039 | **0.0058** |
| Shenque group *vs* Dazhui group | | |  | *χ^2^*=2.6071 | 0.1064 |
| **Recovery rate at day 7** | 478(94.47) | 28(96.55) | 286(87.46) | Fisher | **0.0012** |
| Chemical group *vs* Shenque group | | |  | *χ^2^*=12.8284 | **0.0003** |
| Chemical group *vs* Dazhui group | | |  | Fisher | 0.2274 |
| Shenque group *vs* Dazhui group | | |  | Fisher | 1.0000 |
| **Recovery time (day)** | 3.0(2.0,5.0) | 3.0(2.0,4.0) | 4.0(3.0,6.0) | 25.4792 | <.0001 |
| Chemical group *vs* Shenque group | | |  | 16.4477 | <.0001 |
| Chemical group *vs* Dazhui group | | |  | 18.2069 | <.0001 |
| Shenque group *vs* Dazhui group | | |  | 4.9532 | 0.0260 |
| **Time to return normal temperature (day)** | 2.00(1.00,2.00) | 1.00(1.00,2.00) | 2.0(2.0,4.0) | 25.8445 | <.0001 |
| Chemical group *vs* Shenque group | | |  | 22.7930 | <.0001 |
| Chemical group *vs* Dazhui group | | |  | 10.6999 | 0.0011 |
| Shenque group *vs* Dazhui group | | |  | 1.1811 | 0.2771 |

| Table S10. Analysis of clinical recovery of upper respiratory tract infection in subgroup analysis-7 (%) | | | | | | | |
| --- | --- | --- | --- | --- | --- | --- | --- |
|  | Yin Hu Gan Mao San only | | | | Chemical group (n=327) | Statistic | *P* value |
|  | Shenque group (n=251) | | Dazhui group  (n=14) | |  |  |  |
| **Recovery rate at day 3** | 130(51.79) | | 8(57.14) | | 118(36.09) | *χ^2^*=15.4031 | **0.0005** |
| Chemical group *vs* Shenque group | | | | |  | *χ^2^*=14.3015 | **0.0002** |
| Chemical group *vs* Dazhui group | | | | |  | *χ^2^*=2.5552 | 0.1099 |
| Shenque group *vs* Dazhui group | | | | |  | *χ^2^*=0.1521 | 0.6966 |
| **Recovery rate at day 5** | 194(77.29) | | 13(92.86) | | 225(68.81) |  |  |
| Chemical group *vs* Shenque group | | | | |  | *χ^2^*=5.1249 | 0.0236 |
| Chemical group *vs* Dazhui group | | | | |  | Fisher | 0.0727 |
| Shenque group *vs* Dazhui group | | | | |  | Fisher | 0.3157 |
| **Recovery rate at day 7** | 241(96.02) | | 14(100.00) | | 286(87.46) | Fisher | **0.0007** |
| Chemical group *vs* Shenque group | | | | |  | *χ^2^*=12.9159 | **0.0003** |
| Chemical group *vs* Dazhui group | | | | |  | Fisher | 0.3901 |
| Shenque group *vs* Dazhui group | | | | |  | Fisher | 1.0000 |
| **Recovery time (day)** | | 3.0(2.0,5.0) | | 3.0(2.0,4.0) | 4.0(3.0,6.0) | 12.4348 | 0.0020 |
| Chemical group *vs* Shenque group | | | | |  | 7.7593 | 0.0053 |
| Chemical group *vs* Dazhui group | | | | |  | 7.4024 | 0.0065 |
| Shenque group *vs* Dazhui group | | | | |  | 2.3572 | 0.1247 |
| **Time to return normal temperature (day)** | | 2.00(1.00,3.00) | | 1.00(1.00,2.50) | 2.0(2.0,4.0) | 10.4971 | 0.0053 |
| Chemical group *vs* Shenque group | | | | |  | 9.2845 | 0.0023 |
| Chemical group *vs* Dazhui group | | | | |  | 2.6917 | 0.1009 |
| Shenque group *vs* Dazhui group | | | | |  | 0.3910 | 0.5318 |

| Table S11. Analysis of clinical recovery of upper respiratory tract infection in subgroup analysis-8 (%) | | | | | |
| --- | --- | --- | --- | --- | --- |
|  | Yin Hu Gan Mao San combined | | Chemical group (n=327) | Statistic | *P* value |
|  | Shenque group (n=255) | Dazhui group  (n=15) |  |  |  |
| **Recovery rate at day 3** | 141(55.29) | 13(86.67) | 118(36.09) | *χ^2^*=31.7950 | **<.0001** |
| Chemical group *vs* Shenque group | | |  | *χ^2^*=21.4040 | **<.0001** |
| Chemical group *vs* Dazhui group | | |  | *χ^2^*=15.5270 | **<.0001** |
| Shenque group *vs* Dazhui group | | |  | *χ^2^*=5.6901 | 0.0171 |
| **Recovery rate at day 5** | 217(85.10) | 14(93.33) | 225(68.81) | Fisher | **<.0001** |
| Chemical group *vs* Shenque group | | |  | *χ^2^*=20.8133 | **<.0001** |
| Chemical group *vs* Dazhui group | | |  | Fisher | 0.0455 |
| Shenque group *vs* Dazhui group | | |  | Fisher | 0.7041 |
| **Recovery rate at day 7** | 237(92.94) | 14(93.33) | 286(87.46) | Fisher | 0.0780 |
| Chemical group *vs* Shenque group | | |  | *χ^2^*=11.6752 | **0.0006** |
| Chemical group *vs* Dazhui group | | |  | Fisher | 0.6221 |
| Shenque group *vs* Dazhui group | | |  | Fisher | 1.0000 |
| **Recovery time (day)** | 3.0 (3.0,5.0) | 3.0 (2.0,3.0) | 4.0 (3.0,6.0) | 25.0740 | <.0001 |
| Chemical group *vs* Shenque group | | |  | 17.5717 | <.0001 |
| Chemical group *vs* Dazhui group | | |  | 12.7033 | 0.0004 |
| Shenque group *vs* Dazhui group | | |  | 3.7912 | 0.0515 |
| **Time to return normal temperature (day)** | 2.00 (1.00,2.00) | 1.00 (1.00,2.00) | 2.0 (2.0,4.0) | 31.3699 | <.0001 |
| Chemical group *vs* Shenque group | | |  | 28.2994 | <.0001 |
| Chemical group *vs* Dazhui group | | |  | 9.2194 | 0.0024 |
| Shenque group *vs* Dazhui group | | |  | 0.6303 | 0.4272 |

| Table S12. Adverse events during observation period | | | | | | | | | | | | |
| --- | --- | --- | --- | --- | --- | --- | --- | --- | --- | --- | --- | --- |
| No. | Group | Description | Start | End | Severity | SAE | Prognosis | Measures to Yin Hu Gan Mao San | Target treatment | Drop out | Relationship about Yin Hu Gan Mao San | AE related to Yin Hu Gan Mao San |
| 2011 | Exposed | Erythema at application site (umbilical cord) | 2022/9/9 | 2022/9/9 | Mild | Not | Resolved | Continued use | Not | Not | Possibly Related | Yes |
| 2017 | Exposed | Erythema, pruritus and rash at application site | 2022/9/20 | 2022/9/23 | Mild | Not | Resolved | Dosage reduction | Not | Not | Possibly Related | Yes |
| 2020 | Exposed | Erythema, pruritus and rash at application site (umbilical cord and Dazhui) | 2022/10/2 | 2022/10/5 | Mild | Not | Resolved | Dosage reduction | Not | Not | Definitely Related | Yes |
| 6004 | Exposed | Iron deficiency anemia (Hb 95 g/L) | 2022/6/20 |  | Mild | Not | Persisted | Dosage reduction | Not | Not | Possibly Unrelated | Not |
| 12006 | Exposed | Pruritus at application site | 2022/6/10 | 2022/6/15 | Mild | Not | Resolved | Dosage reduction | Not | Not | Possibly Related | Yes |
| 13019 | Exposed | Rash at application site (umbilical cord) | 2022/7/20 | 2022/7/21 | Mild | Not | Resolved | Drug withdrawal | Not | Not | Possibly Related | Yes |
| 17046 | Exposed | Acute conjunctivitis at left | 2022/9/15 | 2022/9/19 | Mild | Not | Resolved | Continued use | Tobramycin drops in the left once an hour | Not | Possibly Unrelated | Not |
| 21004 | Exposed | Erythema at application site | 2022/9/16 | 2022/9/17 | Mild | Not | Resolved | Drug withdrawal | Not | Not | Possibly Related | Yes |
| 21010 | Exposed | Pruritus | 2022/9/21 | 2022/9/23 | Mild | Not | Resolved | Drug withdrawal | Not | Not | Possibly Related | Yes |
| 21021 | Exposed | Pruritus and rash | 2022/9/29 | 2022/9/30 | Mild | Not | Resolved | Drug withdrawal | Not | Not | Highly Likely Related | Yes |
| 21026 | Exposed | Erythema at application site | 2022/10/9 | 2022/10/16 | Mild | Not | Resolved | Continued use | Not | Not | Possibly Related | Yes |
| 21036 | Exposed | Erythema at application site | 2022/10/20 | 2022/10/23 | Mild | Not | Resolved | Drug withdrawal | Not | Not | Possibly Related | Yes |
| 24002 | Exposed | Otitis media | 2022/8/3 | 2022/8/14 | Mild | Not | Resolved | Drug withdrawal | Not | Not | Possibly Unrelated | Not |
| 24013 | Exposed | Roseola Infantum | 2022/8/4 | 2022/8/7 | Mild | Not | Resolved | Drug withdrawal | Not | Not | Possibly Unrelated | Not |
| 24020 | Exposed | Roseola Infantum | 2022/8/7 | 2022/8/14 | Mild | Not | Resolved | Drug withdrawal | Not | Not | Possibly Unrelated | Not |
| 24022 | Exposed | Roseola Infantum | 2022/8/7 | 2022/8/11 | Mild | Not | Resolved | Drug withdrawal | Not | Not | Possibly Unrelated | Not |
| 24022 | Exposed | Diarrhea | 2022/8/7 | 2022/8/11 | Mild | Not | Resolved | Drug withdrawal | Not | Not | Possibly Unrelated | Not |
| 25017 | Non-Exposed | Acute bronchitis | 2022/8/20 | 2022/8/27 | Mild | Not | Resolved | Drug withdrawal | Not | Not | Possibly Unrelated | Not |
| 25069 | Exposed | Bronchopneumonia | 2022/11/8 | 2022/11/13 | Mild | Not | Resolved | Drug withdrawal | Not | Not | Possibly Unrelated | Not |
| 26009 | Exposed | Viral rash | 2022/9/21 | 2022/9/28 | Mild | Not | Resolved | Drug withdrawal | Not | Not | Possibly Unrelated | Not |
